# Recognized and Emerging Features of Erythropoietic and X-Linked Protoporphyria

**DOI:** 10.3390/diagnostics12010151

**Published:** 2022-01-08

**Authors:** Elena Di Pierro, Francesca Granata, Michele De Canio, Mariateresa Rossi, Andrea Ricci, Matteo Marcacci, Giacomo De Luca, Luisa Sarno, Luca Barbieri, Paolo Ventura, Giovanna Graziadei

**Affiliations:** 1 Dipartimento di Medicina Interna, Fondazione IRCCS Cà Granda Ospedale Maggiore Policlinico, 20122 Milan, Italy; francesca.granata@policlinico.mi.it (F.G.); giacomodeluca29@gmail.com (G.D.L.); giovanna.graziadei@policlinico.mi.it (G.G.); 2 Porphyria and Rare Diseases Centre, San Gallicano Dermatological Institute IRCCS, 00144 Rome, Italy; michele.decanio@ifo.gov.it (M.D.C.); luca.barbieri@ifo.gov.it (L.B.); 3 Department of Dermatology, ASST Spedali Civili di Brescia, University of Brescia, 25123 Brescia, Italy; dottoressarossimt@gmail.com (M.R.); luisa.sarno0@gmail.com (L.S.); 4 Internal Medicine Unit, Department of Medical and Surgical Science for Children and Adults, University of Modena e Reggio Emilia, 41124 Modena, Italy; andrewrk92@gmail.com (A.R.); marcacci.matteo@aou.mo.it (M.M.); paoloven@unimore.it (P.V.)

**Keywords:** erythropoietic protoporphyria, X-linked protoporphyria, phototoxicity, liver disease, anemia, inflammation, osteoporosis

## Abstract

Erythropoietic protoporphyria (EPP) and X-linked protoporphyria (XLP) are inherited disorders resulting from defects in two different enzymes of the heme biosynthetic pathway, i.e., ferrochelatase (FECH) and delta-aminolevulinic acid synthase-2 (ALAS2), respectively. The ubiquitous FECH catalyzes the insertion of iron into the protoporphyrin ring to generate the final product, heme. After hemoglobinization, FECH can utilize other metals like zinc to bind the remainder of the protoporphyrin molecules, leading to the formation of zinc protoporphyrin. Therefore, FECH deficiency in EPP limits the formation of both heme and zinc protoporphyrin molecules. The erythroid-specific ALAS2 catalyses the synthesis of delta-aminolevulinic acid (ALA), from the union of glycine and succinyl-coenzyme A, in the first step of the pathway in the erythron. In XLP, ALAS2 activity increases, resulting in the amplified formation of ALA, and iron becomes the rate-limiting factor for heme synthesis in the erythroid tissue. Both EPP and XLP lead to the systemic accumulation of protoporphyrin IX (PPIX) in blood, erythrocytes, and tissues causing the major symptom of cutaneous photosensitivity and several other less recognized signs that need to be considered. Although significant advances have been made in our understanding of EPP and XLP in recent years, a complete understanding of the factors governing the variability in clinical expression and the severity (progression) of the disease remains elusive. The present review provides an overview of both well-established facts and the latest findings regarding these rare diseases.

## 1. Introduction

Protoporphyria (PP) is a rare genetic metabolic disorder caused by inborn defects in enzymes of the heme-biosynthetic pathway. The first comprehensive description of the disease was published by Magnus et al. in 1961 [1]. Two clinically indistinguishable forms are recognized: erythropoietic protoporphyria (EPP, MIM 177000) and X-linked protoporphyria (XLP, MIM 300752). EPP results from the deficiency of ferrochelatase (FECH, EC 4.99.1.1), the final enzyme in the heme-biosynthetic pathway [2]. XLP, a less common condition, results from the genetically upregulated activity of the erythroid-specific aminolevulinic acid synthase (ALAS2, EC 2.3.1.27), the first enzyme of the heme-biosynthetic pathway in the erythroid cells [3].

EPP is an autosomal recessive condition [4] that occurs because of a loss-of-function mutation in one allele of the *FECH* gene, which is mostly accompanied by a low expression *FECH* genetic variant (c.315–48C) in the other allele, leading to a 70% decrease in the enzyme activity [5]. In a recent paper, it has been shown that this variant is linked to a single haplotype encompassing the first 20 Kb of the *FECH* gene. Among all the variants included in this haplotype, association analysis has revealed the GTC haplotype to be the most common (c.1–252G, c.68–23T, c.315–48C), with TC haplotype occurring in only one case [6]. In the same paper, the authors concluded that the c.315–48 C variant in isolation is necessary but not sufficient to cause an overt disease even when inherited in homozygosis. Rarely, patients can inherit bi-allelic loss-of-function mutations in the *FECH* gene, which leads to a more severe form of the disease and accounts for about 4% of the cases in Europe. In these patients, the majority of the mutations are missense and inherited in compound heterozygosity [7].

XLP occurs due to gain-of-function mutations that are localized in the terminal exon of the X-linked *ALAS2* gene [8]; this region encodes for the carboxyl-terminal portion of the enzyme, which is crucial for its activity and stability [9]. All male carriers are affected; however, in heterozygous females with XLP, the random X-inactivation pattern directly influences the penetrance and the severity of the phenotype [10]. Recently, an autosomal dominant mutation in the *CLPX* gene, a modulator of ALAS2 activity, was also reported [11].

EPP and XLP cause the accumulation of protoporphyrin molecules in the circulating erythrocytes and plasma, which are subsequently taken up by the liver and vascular endothelium, including the superficial skin vasculature [12,13]. The protoporphyrin molecules are photodynamic and absorb light radiation of the visible blue-violet light in the Soret band and the long-wave UV region (to a lesser degree) [14]. When exposed to sunlight, they are photoactivated, which triggers singlet oxygen-mediated radical reactions, leading to tissue and vessel damage, activation of the complement system, and release of histamines and chemotactic factors [15].

**Figure 1:**
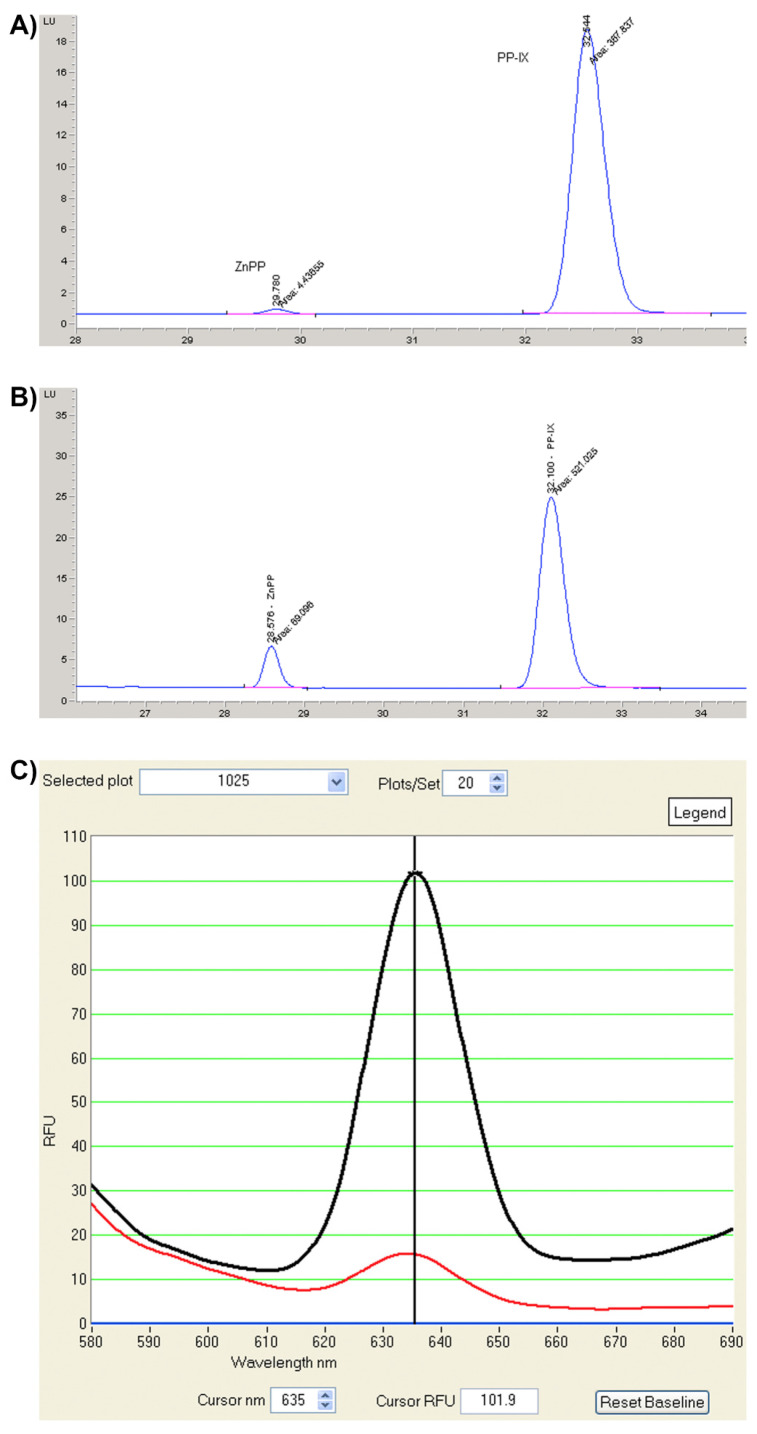
Protoporphyrin detection. (**A**) Chromatographic profile of erythrocyte porphyrins from an EPP patient. (**B**) Chromatographic profile of erythrocyte porphyrins from an XLP patient. (**C**) Plasma fluorimetric emission scanning at high (black line ) and low (red line) concentrations. PPIX: metal-free protoporphyrin IX and ZnPP: zinc protoporphyrin.

EPP and XLP are diagnosed based on the biochemical confirmation of total erythrocyte protoporphyrin (ePP), metal-free protoporphyrin IX (PPIX), and zinc bound protoporphyrin (ZnPP) [16]. FECH deficiency in EPP leads to high ePP levels that are predominantly PPIX (85% to 100%) with a low amount of ZnPP (Figure 1A). In contrast, XLP, with preserved FECH and increased ALAS2 activity, leads to an increase in the PPIX (50% to 85%) and ZnPP levels (Figure 1B). Plasma protoporphyrin molecules are also elevated, with a characteristic fluorescent peak from 632 to 636 nm (Figure 1C); however, urinary protoporphyrin levels are normal [17]. EPP has been described in patients worldwide, with an estimated prevalence ranging from 1:75,000 in the Netherlands to 1:200,000 in the United Kingdom [18]. Among these XLP accounts for about 2–5% of the cases in Europe [19,20] and approximately 10% of the cases in the United States [8].

## 2. Cutaneous Manifestations

Protoporphyria (PP) presents itself as acute, painful phototoxicity on sun-exposed areas starting at infancy or childhood [21]. The mean age of the onset of symptoms is four years [13]. However, often a wide discrepancy is reported between the common median age of disease onset and the diagnosis (frequently in the adult age) due to the rarity of the disease and the variability of symptoms [22]. Late-onset cases, with PP first manifesting after 40 years of age, have been described in association with myelodysplastic syndrome, although cases without this association have also been detected [23]. Photosensitivity manifests in early spring, summer, and early autumn. Poh-Fitzpatrick [24] described the ‘priming phenomenon’, according to which, first sun exposure results in a moderate reaction that might be followed by severe long-lasting reactions during subsequent days if exposure is repeated even for a very short time. Patient photosensitivity might also be aggravated by heat exposure or temperature gradients [25].

**Figure 2:**
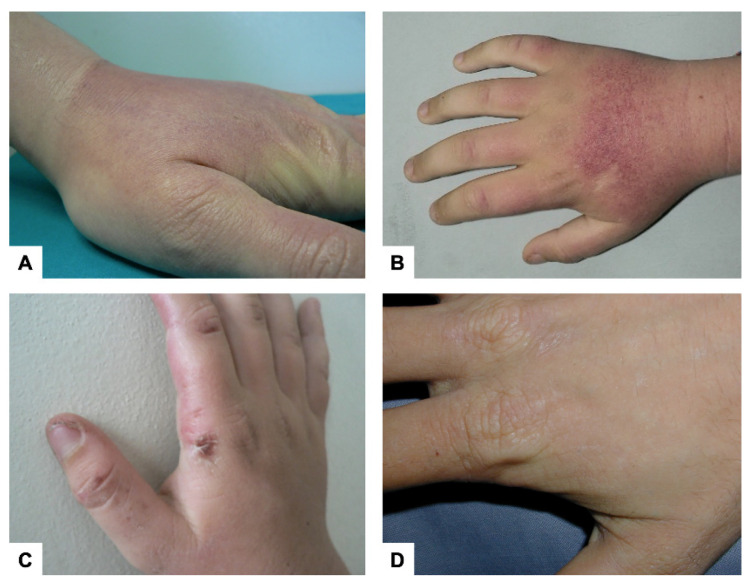
Cutaneous manifestations. (**A**) Edema. (**B**) Erythema. (**C**) Vesicles and bullous lesions. (**D**) Scarring of sun exposed areas.

The clinical presentation in males and females is similar. Any sun-exposed area can be affected; however, the back of the hands and face are most commonly involved. The pain is usually preceded by tingling, itching, and burning sensations of the skin, occurring within minutes of sun exposure in autumn and winter as well. Patients can develop erythema and edema of the sun-exposed skin (Figure 2A,B). PP may also be manifested as solar urticaria. Vesicles or bullous lesions are uncommon in this disorder, but rarely, patients may present these symptoms (Figure 2C) in a rather acute form that distinguishes them from other cutaneous porphyria conditions, where they appear after a longer delay [13]. In a study, blistering was self-reported by about 26% of the patients [26]. Multiple episodes of acute photosensitivity may lead to chronic changes of sun-exposed skin (lichenification, loss of lunulae of the fingernails, ecchymoses, petechiae, and minor scarring on the face and vertical grooving of the lips) (Figure 2D) [27]. Severe scarring, hypo or hyperpigmentation, skin friability, and hirsutism are not typically observed [13]. Palmar keratoderma has been reported in some individuals with two loss-of-function *FECH* mutations [28].

In PP patients, the skin is more sensitive to longer ultraviolet wavelengths (UVA) and visible light [29]; the wavelength of light that causes skin reactions in PP, unlike those that cause sunburn (UVB), can pass through window glass. Studies have reported that UVA phototest positivity is associated with high PPIX concentration [30]. Clinical manifestations are highly variable, with some patients unable to tolerate even a few minutes of sun exposure and some able to tolerate several hours of sun exposure. Most patients develop symptoms within 30 min of sun exposure. The pain is severe, and the symptoms may seem out of proportion to the skin lesions or the lack of them. Recovery from symptoms may take up to 4–7 days. Symptoms may also vary based on environmental conditions, including season, cloud cover, the intensity and extent of sun exposure, and the time of the day. With time, most patients can recognize the prodromal symptoms of PP (e.g., itching and tingling) which serve as a warning sign to seek shade. Patients also develop a conditioned behavior of sun avoidance by using protective clothing, shade, and tinted windows. This might impact their daily activities, such as missing school or work [13].

Histological examination of acute lesions shows epidermal vacuolization and intercellular edema in addition to endothelial vacuolization and cytolysis in the superficial blood vessels of the skin, without any other alterations of the surrounding dermis. Progressively, the deposition of PAS1 hyaline material around dermal blood vessels results in the thickening of capillary basement membranes. Electron microscopic examination of chronically exposed skin revealed progressive reduplication of the basement membrane of the superficial vessel walls, which is caused by repeated endothelial photoinduced damage. These lesions result from a photodynamic oxygen-dependent reaction, where protoporphyrin deposited on the skin that diffuses from the dermal vasculature acts as the chromophore [25]. Differential diagnoses include phototoxic drug reactions, hydroa vacciniforme, solar urticaria, contact dermatitis, angioedema, and lupus erythematosus. Chronic lesions should be differentiated from lipoid proteinosis [12].

## 3. Liver Involvement

Even though the cutaneous involvement of PP is the most debilitating condition for the majority of the patients, a small but significant percentage of the patients suffer from liver disease due to progressive deposition and accumulation of insoluble PPIX in hepatocytes and bile canaliculi [31]. PPIX is usually excreted into the bile and a part of this is absorbed by the intestine and returned to the liver as part of the enterohepatic circulation [32]. Excess PPIX can crystallize, forming stones and exerting a cholestatic effect on the liver, which leads to architectural changes in the hepatobiliary system that range from mild inflammation to fibrosis and cirrhosis [33]. Liver injury causes impairment of PPIX biliary excretion, resulting in a vicious cycle of further PPIX accumulation in plasma and increased PPIX-mediated toxicities. PP patients can also develop severe abdominal pain along with back pain and proximal motor neuropathy due to liver injury [34]. This so-called protoporphyric hepatopathy can be a serious complication of PP, potentially leading to acute and rapidly progressive liver failure and the need for liver transplantation [35].

Four degrees of hepatobiliary disease in patients with EPP have been described: cholelithiasis in young individuals (formation of biliary gallstones), mild parenchymal liver disease (elevation of necrosis and stasis indices), progressive hepatocellular disease, and acute cholestatic liver failure [36,37].

Approximately 5–20% of PP patients show clinical symptoms of liver disease, with hepatic failure occurring in 1–5% of the patients [36,37]. Through abdominal ultrasonography and transient elastography in a cohort of 114 adult patients, a recent study determined the prevalence of liver steatosis (29%) and liver stiffness (9.6%). Although these conditions occur at a similar rate in the general population, liver injury in PP patients might be correlated with increased erythrocyte PPIX levels.

Moreover, the same study confirmed a high prevalence of cholelithiasis (25.7%), while abnormal levels of liver enzymes (transaminases, alkaline phosphatase and γ-glutamyl transferase) were found in 6.2% of the PP patients [38]. However, normal liver function tests have also been reported in patients with PP-related fibrosis and cholestasis [39].

Even in early PP, ultrastructural damage associated with protoporphyrin crystals has been described in hepatocyte nuclei, endoplasmic reticulum, plasma membranes, and bile canaliculi [40]. Fluorescent birefringence studies have revealed the presence of significant amounts of intracellular protoporphyrin precipitates in liver samples of PP patients [37,41]. A mild accumulation of α1-antitrypsin has also been reported in Kupffer cells, but not in hepatocytes. However, no hemosiderin or copper was detectable by special stains [42]. A recent study demonstrated the unique property of porphyrins to cause organelle-selective protein oxidation and aggregation, which is thought to be a major mechanism of cellular injury in porphyria [43]. As internal organs are not exposed to light, photosensitized porphyrin-protein aggregation is not applicable; thus, an alternative porphyrin-mediated cellular damage was proposed, at least for hepatocytes [44]. In this study, the authors reported that a light-independent porphyrin-mediated protein aggregation occurs in internal organs after secondary triggers of oxidative stress, such as inflammation or other injuries. This, in turn, leads to the formation of ROS and protein oxidation. Porphyrins then bind to the oxidized proteins further promoting protein aggregation.

Animal studies have shown that excessive PPIX exposure can result in periportal or septal fibrosis accompanied by an atypical ductular reaction [45]. Paradoxically, an unimpaired bile flow was reported and biliary bile salt secretion was four times higher in EPP mice than in controls. Moreover, there was a two-fold increase in the bile salt/lipid ratio [46]. PPIX binds to bile components and disrupts the physiological equilibrium of phospholipids, bile acids, and cholesterol present in bile. Some studies have proposed that PPIX retention in hepatocytes and Kupffer cells with a consequent lower biliary PPIX efflux and the formation of less lipid-free bile acid could prevent the development of liver complications [47].

The precise mechanisms responsible for liver failure are not fully understood; however, it has been hypothesized that the ABCG2-dependent delivery of PPIX to the biliary system, causing bile duct blockage, could play an important role [48]. The authors of this paper demonstrated that ABCG2 transporter deficiency could protects against PP-associated hepatotoxicity by modulating PPIX distribution, metabolism, and excretion. Moreover, the decrease of PPIX distribution on the skin also prevents PP-associated phototoxicity.

Since there is no clear relationship between the genetic defect responsible for PP and hepatic accumulation of PPIX, a few researchers have proposed that additional genetic factors might be involved in the pathogenesis of this rare complication [49]. However, the identification of the factors determining the individual predisposition to liver involvement is still an active area of research. The loss of the porphyrin transporter ABCB6 was reported to exacerbate liver damage in a Fech-deficient mouse model with extensive deposits of protoporphyrin crystals in hepatocytes, signs of parenchymal disarray, higher total serum bilirubin levels, and increased levels of markers of liver injury and inflammation. In the same study, an EPP patient with fatal liver failure harbored a common variant of ABCB6 (T521S) that was found by in vitro experiments to cause the loss of the protein [50]. Due to a small cohort of analyzed patients, the potential contribution of candidate transporters in the development of PP-associated liver disease needs further investigation.

## 4. Anemia

A mild hypochromic, microcytic anemia has been reported in 33% of men and 48% of women with PP [51]. However, the presence of iron deficiency remains unclear. A two-thirds decrease in the iron stores (assessed by serum ferritin) of PP patients was observed. However, soluble transferrin receptor-1 and iron concentrations in serum were reported to be normal, suggesting that erythropoiesis is not limited by iron supply [51,52]. Moreover, an inappropriate increase was not observed in the hepcidin level in both serum and urine, thus excluding a decreased enteral iron absorption [53]. Finally, the iron deficiency in these disorders is not related to chronic inflammation or iron loss [54]. A positive correlation between erythrocyte PPIX and serum transferrin (Tf) level was described in a mouse model of EPP [55], suggesting a higher mobilization of tissue iron stores to meet the requirement of erythropoiesis. Studies have shown a negative correlation of PPIX with iron and Tf saturation in PP patients, leading to the conclusion that PPIX stimulates the hepatic synthesis of Tf and influences distribution of the iron in peripheral iron stores such as the spleen, and the bone marrow [52]. However, a protective role of iron deficiency cannot be completely excluded [56].

## 5. Vitamin D Deficiency and Osteoporosis

Vitamin D belongs to the category of fat-soluble vitamins and can promote the absorption of calcium and phosphorous, thereby influencing the bone mineralization. However, in the last decade, various other functions of vitamin D have been reported. Vitamin D is measured in the body by determining the levels of 25-hydroxycholecalciferol (25[OH]D) and values below 20 ng/mL (50 nmol/L) are considered inadequate [57]. The optimal serum concentration of 25(OH)D must exceed 30 ng/mL. Since sunlight is the major source of vitamin D, lower latitude and higher intensity of sunlight have been associated with a lower risk of vitamin D deficiency [58,59,60]. Assuming that PP patients avoid the sun, it is important to assess the concentration of vitamin D to prevent bone damage and other health problems linked to its deficiency.

In an uncontrolled study, the vitamin D level of 201 PP patients was analysed. The researchers collected the samples for seven months, i.e., between January and July, in order to monitor the levels in the winter and summer seasons. In general, low levels of vitamin D were detected in both seasons, with a slight increase in summer. The mean of serum 25(OH)D increased for every month in the observation time, from 15.5 to 21.3 ng/mL. Overall, results indicated that 34 patients (17%) were deficient and 126 (63%) had insufficient levels of vitamin D [61].

Similar results were obtained in a cross-sectional study on 48 Dutch PP patients during the period between June and November. Twenty-two patients (46%; 15 males and 7 females) were deficient in vitamin D, and there was a significant difference in mean serum 25(OH)D between female and male patients [62].

A longitudinal controlled prospective cohort study was conducted in the UK on 53 PP patients and 109 controls across different seasons. The results showed a lack of 25(OH)D during summer and 47% of the PP samples showed a lower level of vitamin D compared to the 18% of the control samples [63].

A lack of vitamin D leads to osteoporosis, and to investigate further this condition, a bone mineral density (BMD) test was performed with four males and six females. The results showed that the lumbar T-score median levels were in the osteopenia range in both female and males [64]. These results were confirmed in a study performed with 44 PP patients. Osteopenia was present in 36% and osteoporosis in 23% of the patients [65].

## 6. Systemic Inflammation

The biological process that leads to a phototoxic reaction in PP patients has not been completely elucidated. Thunell et al., in 2000, first hypothesized that PPIX excitated at 410 nm leads to singlet oxygen (^1^O_2_*) mediated damage of endothelial cells [66]. The photodamage leads to the stimulation of an inflammatory response, occurring via complement system (CS) activation and mast cell degranulation, with exocytosis of vasoactive mediators. The CS comprises a group of 50 proteins, and as a part of the innate immune system, it can be activated through three different pathways: classical, lectin, and alternative (also known as the properdin pathway) [67]. A few studies based on in vitro experiments in both animal and human serum have focused on the CS activation in porphyria. In both models, the activation of exogenously added uroporphyrin, or protoporphyrin, after exposure to a light source emitting 400–410 nm radiation resulted in the activation of the CS and the generation of chemotactic activity in human polymorphonuclear leukocytes [68,69]. Dosage studies of the most common proteins of CS, i.e., C3 and C5, were conducted in vivo on different kinds of tissues (skin and serum), in a few patients. The first evidence of the activation of the CS was demonstrated by the collection of blood and skin samples after irradiation (0.7 J/cm^2^ at 400–410 nm) in two PP patients [70,71]. Moreover, a study in 1980, conducted on serum samples of 14 photosensitive asymptomatic PP patients before and after UV irradiation, showed marked activation of CS for Cl, C4, C2, and C3 proteins after UV irradiation; also, the levels of C3 correlated with the concentration of PPIX in the plasma [72].

Several studies in the last decade have focused on the function of the CS in inflammation, especially in chronic diseases (arthritis, asthma, and kidney failure). Active participation of this pathway has been well-established in regulating the homeostasis of tissues, eradicating cellular debris, orchestrating immune responses, and sending ‘danger’ signals [67,73].

A recent study on 18 PP patients reported a non-invasive method that allowed the analysis of CS fluctuations associated with the variation of light intensity between seasons without exposing the patients to dangerous and painful treatment. The study showed a significant increase in the C3 and factor-B (FB) proteins, primarily in summer, which highlighted the involvement of the alternative pathway (AP) of the CS in the phototoxic reaction of patients [74]. Another study reported an increase in C5 and properdin and a decrease in inhibitor factor H (FH) of the AP in summer [75].

Properdin is the main protein that binds to FB-C3b complex, triggering the amplification loop in the AP. Properdin is positively correlated with C3 in summer, suggesting the stimulation of the AP in summer. All assumptions were supported by the evaluation of exposure to global solar radiation, collected by the regional agency of environmental protection (ARPA). With an increase in global radiation, the main factors of the AP, such as C3 and properdin, also increase. A decrease in the FH values with a concomitant increase in global radiation, confirmed the loss of inhibition of the AP loop in summer [75].

These results confirmed the presence of inflammation in the phototoxic reaction and explained the chills, malaise, fatigue, nausea, excessive temperature sensitivity, and general sickness reported by patients after long solar exposure. These symptoms could be attributed to the functions of the systemic immune system [31,76].

## 7. Management

### 7.1. Photoprotection

The prevention of phototoxic reactions due to the activation of PPIX by sunlight forms the basis of PP management. The majority of the patients learn to manage their disease by avoiding sun exposure after the appearance of prodromal symptoms. However, prolonged exposure to sunlight leads to more severe symptoms, such as pain that does not respond to narcotic analgesics and may last for several days. Some patients find relief by applying cold wet compresses to the affected areas or by using fans or air conditioners. The recurrence of these events establishes a lifestyle that is oriented toward avoiding sunlight, leading to negative consequences on social life, as well as on the quality of life (QoL). The conventional topical sunscreens are not effective in preventing phototoxic reactions due to poor protection in the longer UVA and light in the visible wavelength. Sunscreens containing zinc oxide or titanium dioxide in addition to an absorbing pigment can block the visible light, but they might be unpleasant aesthetically [77]. Patients are used to wearing photoprotective clothing, such as long-sleeved dresses, gloves, wide-brimmed hats, and sunglasses. Photoprotection measures, such as the application of yellow filters to lights in the operating room, must be considered in the case of long-lasting surgeries that expose large surfaces of the skin and internal organs to phototoxic damage [78]. This is particularly relevant for those PP patients who have high erythrocyte PPIX levels and are undergoing abdominal surgery, such as liver transplantation for cholestatic disease. Polyimide filters offer the possibility of operating safely with adequate visibility [79].

### 7.2. Photosensitivity

In the past, a variety of pharmacological products were tested to minimize the pathogenic effect of PPIX by increasing skin coloration and enhancing antioxidant defenses against free radicals. Oral β-carotene, administered at high dosages, can cause mild yellowish skin pigmentation, leading to several studies focusing on its potential photoprotective effect, but with contradictory results. Minder et al. critically reviewed these studies highlighting their methodological shortcomings, as most of the works were open-label studies, uncontrolled studies, or retrospective case reports [80]. There was only one randomized, crossover-controlled trial [81], whose results indicated no effect or negative effect of exposure time to bright sunlight in nine out of 11 patients. The study was performed with an overall minimal increase in the mean exposure time per day, and the same authors evaluated the results to be clinically irrelevant. A drawback of using β-carotene is that its dosage should not exceed 25 mg/day, as higher dosages entail an increased risk of lung and stomach cancer [82]. More effective therapy in inducing skin pigmentation is based on the stimulation of melanocytes by UV radiation [15]. Phototherapy protocols have been performed by using narrow band-UVB [83,84] or psoralen-UVA [85]; however, only a few EPP patients were effectively treated. A major concern is the increased risk of developing skin cancers after several treatment sessions. Other therapeutic options tested to ameliorate photosensitivity in EPP patients include the oral intake of N-acetyl-cysteine [86,87], cysteine [88,89], and vitamin C [90], but the experimental data does not support the clinical efficacy of these treatments [80]. Nevertheless, in clinical practice, antioxidants, such as β-carotene (provitamin A) and vitamin C, are usually prescribed as therapeutic measures to mitigate the photosensitivity in PP patients. It is commonly believed that these patients must increase their intake of antioxidants to counteract the oxidative cellular damage caused by the chronic accumulation of PPIX in erythrocytes and the liver. In a recent pilot study [91], Parker et al. tested isoniazid that is known to inhibit ALAS2 activity, the rate limiting enzyme of heme biosynthesis in erythroid cells. Although the results obtained in the animal model were promising, isoniazid, with a dosage of up to 300 mg, was unable to decrease serum and erythrocyte PPIX levels in 15 tested patients.

Presently, afamelanotide (Scenesse, Clinuvel Pharmaceuticals) is a clinically tested treatment proven to improve photosensitivity in PP patients. Results from three clinical trials with two randomized phases demonstrated a longer pain-free duration of exposure to the sun and an improved QoL in afamelanotide-treated patients [92]. The drug, with common adverse reactions such as nausea, fatigue, and headache, is considered to be safe. Afamelanotide was approved for the symptomatic treatment of PP by major drug regulatory authorities, such as the European Medicines Agency (EMA) in December 2014, the US Food and Drug Administration (FDA) in October 2019, and the Australian Therapeutic Goods Administration (TGA) in October 2020. The more recent updates came from the post-authorization observational studies, which followed the marketing approval by EMA [93]. In particular, QoL scores of EPP patients rose after initiating afamelanotide and were stable during the entire treatment period [94,95]; however, the scores decreased rapidly after the interruption of afamelanotide [96]. Although the decrease in the number of phototoxic reactions during the treatment period was not confirmed, these events were perceived as less painful [95,96]. Patients treated with afamelanotide spent more time outside, 6 h or more per week on average [95]. The maximum time spent in sunlight without a phototoxic reaction, defined as phototoxic burn tolerance time (PBTT), increased from the median value of 10 min to 180 min [96]. However, this evidence of efficacy might be biased due to the reluctance of EPP patients to expose themselves to the sun. It was recently proposed that time to prodrome (TTP), i.e., the exposition time until the appearance of prodromal symptoms, could be a more reliable parameter to measure the pharmacological response as safer for patients [97]. Afamelanotide is an analog of the α melanocyte-stimulating hormone (α-MSH) and binds to the melanocortin 1 receptor (MC1R), stimulating the production of eumelanin [98], which provides photoprotection and antioxidant defence in melanocytes [99]. Moreover, due to its anti-inflammatory properties, it was tested for the treatment of various immune-mediated inflammatory disorders [100], such as solar urticaria [101], Hailey–Hailey disease [102], and vitiligo [103]. As recommended by the EMA, it is administered as a biodegradable subcutaneous implant (16 mg) every 60 days and a maximum of four implants per year are allowed [93]. A new emerging therapy, the synthetic molecule MT-7117 (Dersimelagon, Mitsubishi Thanabe Pharma America), has been developed for oral administration. Like afamelanotide, MT-7117 acts through the selective activation of the MCR1 dermal receptor, which induces skin pigmentation due to melanin production. Recently, a Phase II clinical trial was completed for MT-7117, after which, a multicenter, randomized Phase III trial was initiated. Previous results demonstrated increments of more than 50 min in the average time of daily exposure to sunlight without prodromal symptoms in both treatment groups—low dose (100 mg/day) and high dose (300 mg/day). The drug is well tolerated as the commonly reported adverse events are nausea, ephelides, and skin hyperpigmentation [104].

### 7.3. Liver Disease

The management of the hepatic manifestations of EPP is currently sub-optimal, with the efficacy of various treatments being unestablished. Because of the rarity of the disease, no controlled trials have been conducted to validate a specific approach. An algorithm for monitoring early EPP liver disease and managing early and progressive EPP liver disease has been recently proposed [105]. Periodic monitoring of liver function with blood chemistry tests (complete blood count, international normalized ratio, transaminases, and markers of cholestasis) and a radiologic assessment (e.g., abdominal ultrasound) is recommended every 6 to 12 months, depending on the patient’s clinical history. Special attention should be paid to the elevation of the laboratory markers of cholestasis (bilirubin total and conjugated, γ-glutamyl transferase, and alkaline phosphatase).

In some cases, severe liver dysfunction is induced by exogenous triggering agents [106], thereby necessitating the minimization of exposure to hepatotoxic factors. Patients should be encouraged to avoid alcohol and follow a lifestyle and dietary regimen to prevent the onset of nonalcoholic fatty liver disease (NAFLD)/nonalcoholic steatohepatitis (NASH). They should also be screened for the most common hepatotropic viruses (HAV, HBV, and HCV) and vaccinated against those for which a vaccine is available (HAV and HBV). Hepatotoxic drugs should be administered with caution. Based on the experience of the authors, paracetamol should be avoided if possible and replaced with careful administration of non-steroidal anti-inflammatory drugs (NSAIDs) instead. Vitamin E is sometimes administered as an antioxidant for the liver and has been reported to induce a marked decrease in erythrocyte PPIX concentration when administered intravenously [107].

Protoporphyrin gallstones and biliary sludge formation should be prevented in the patients. A combination of chenodeoxycholic acid and ursodeoxycholic acid is administered to enhance the elimination of PPIX through the biliary system [108,109]. However, they were reported to be ineffective in animal models [110].

Furthermore, cholestyramine, a bile sequestering agent, might potentially reduce the plasma levels of protoporphyrin molecules by interrupting their enterohepatic circulation [111,112] although its effective clinical role is debatable [113]. If jaundice and liver dysfunction do not improve even after increased ursodeoxycholic acid intake, cimetidine and lactulose can also be administered. Lactulose is expected to decrease the enterohepatic circulation of protoporphyrin, inhibiting re-absorption of bile acids, while cimetidine is known to reduce hepatic ALA synthase activity [114]. Another approach involves lowering protoporphyrin plasma concentration during the rapidly progressive phase of the disease to reduce further uptake from liver cells. Hematin administration [115,116,117], plasmapheresis [118,119] and low-density lipoproteins (LDL) apheresis (LDL is a major protoporphyrin carrier) [118] have been variously used. Suppressing the release of erythropoietin from kidney interstitial cells might benefit patients during erythrocyte transfusion at higher hemoglobin thresholds (e.g., 9–10 g/dL), even in the absence of clinical signs of anemia [120,121].

For end-stage chronic liver disease, the medical management of PP includes liver transplantation [35]. More than 50 transplants for PP liver disease have been reported with a survival rate of 47–66% after 10 years of follow-up [34]. Although liver transplantation is effective in restoring liver function in patients with PP, it does not cure the FECH deficit in erythroid cells and does not abate liver exposure to plasma protoporphyrin molecules; therefore, there is a real risk of the recurrence of PP-liver disease after transplantation. The risk can be minimized only through hematopoietic cell transplantation [122], which issometimes performed in a sequential approach after liver transplantation [123]. Probably due to the persistence of biliary excretion of protoporphyrin, patients with PP are more susceptible to biliary complications compared to other liver transplant recipients. In this regard, a Roux loop for the safer drainage of bile has been recommended over duct-to-duct anastomosis [124]. However, growing clinical evidence indicates that bone marrow transplantation can restore liver function without the need for a liver transplantation if the cholestatic liver disease can be stabilised by medical treatment and the fibrosis grade is not advanced [125].

### 7.4. Microcytic Anemia

Hypochromic microcytic anemia is generally mild in PP patients. Clinical evidence indicated that iron therapy in XLP might improve liver damage and photosensitivity in addition to anemia [126]. The conserved FECH activity in XLP converts the toxic PPIX into heme depending on the availability of iron. Further investigation is needed to establish whether replenishing iron stores can actually improve the management of these patients. In EPP patients, oral iron supplementation should be considered only for severe iron deficiency after the assessment of hematological and iron storage parameters [127]. Iron can increase the activity of ALAS and then exacerbate the PPIX accumulation in erythrocytes and liver. Therefore, iron therapy must be carefully monitored with complete blood count, ferritin, transferrin saturation, and liver enzymes [12].

### 7.5. Vitamin D Deficiency

Vitamin D supplementation as the active form of cholecalciferol can prevent the vitamin D deficiency due to sun avoidance [128]. A qualitative study on the behavior and attitude regarding vitamin D knowledge was performed with 19 photosensitive patients, including three patients with EPP. Based on direct counseling for 45 min, it was concluded that low vitamin D status is a significant issue in patients with photosensitivity and must be managed appropriately and effectively; all patients were willing to take vitamin D orally [129].

The benefit of vitamin D intake in PP was tested in 46 Danish patients, demonstrating that follow-up on vitamin D status and recommendations of an adequate diet and supplementation are essential to restore 25(OH)D levels [130] (Table 1).

## 8. Conclusions

**Figure 3:**
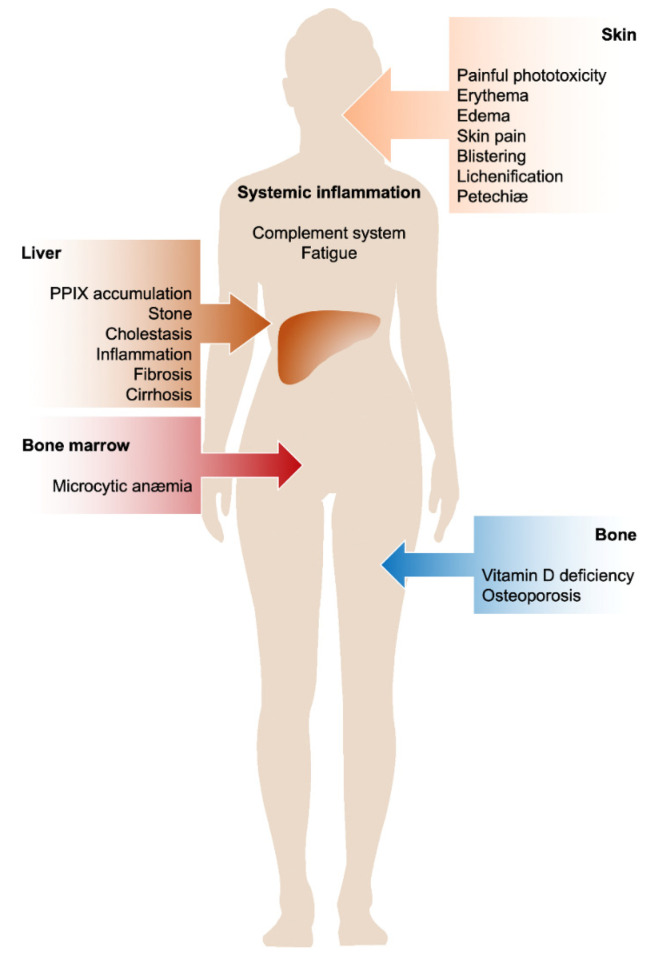
Multi-organ involvement in protoporphyria.

EPP and XLP are known as rare photodermatoses resulting in acute, painful, and non-blistering phototoxicity on sun exposure. However, a wide spectrum of clinical findings indicating hepatobiliary disease have also been reported. The individual susceptibility of patients to protoporphyrin-induced liver damage is highly variable, and the reasons are unknown. Microcytic anemia with abnormal iron metabolism is observed in about 40% of the patients; however, the cause and mechanism of iron deficiency remain to be elucidated. Vitamin D deficiency affects the majority of patients as a consequence of avoiding sunlight. Decreased bone mineral density was also reported in protoporphyria as compared to the general population, indicating the prevalence of osteoporosis and osteopenia in these patients. Finally, the activation of the CS during the phototoxic reaction suggests that a systemic inflammatory response occurs, justifying the general malaise reported by patients after exposure to light for a long time. Based on these observations, EPP and XLP should be considered as chronic metabolic multi-organ disorders and not limited to skin photosensitivity problems or purely dermatological diseases with seasonal onset (Figure 3).

## Figures and Tables

**Table 1:** Management and monitoring of protoporphyria.

Symptoms	Treatment Measurements	Recommendations
Cutaneous Manifestations		
Photoprotection	Protective clothing such as hats, long sleeves, gloves and trousers are beneficial.	Sunscreens mainly protect against UV radiation and do not protect against light that causes EPP symptoms. Windows in houses and cars offer no protection since harmful visible light passes.
	Application of yellow filters to lamps in the operating room	Only in PP patients who are undergoing long-lasting abdominal surgery such as liver transplantation.
Photosensitivity	Afamelanotide (Scenesse^®^)	16 mg subcutaneous implant every 60 days with a maximum of four implants per year
**Liver involvement**		
Gallstones	Ursodeoxycholic acid	Monitoring of erythrocyte protoporphyrins, liver function tests, and necrosis and stasis indices. Abdominal imaging (ultrasound or computed tomography) every 6 to 12 months depending on the patient
Cholestasis	Plasma exchange and erythrocyte transfusion	
Acute liver failure	Liver or bone marrow transplantation	Bone marrow transplantation can restore liver function without the need for a liver transplantation if the cholestatic liver disease can be stabilised by medical treatment and the fibrosis grade is not advanced
**Iron deficiency anemia**	Oral iron supplementation	In EPP patients, oral iron supplementation should be considered only for severe iron deficiency and not just routinely when the patient is slightly anemic. Monitoring of complete blood count, ferritin, transferrin saturation and erythrocyte protoporphyrins every 6 months.
**Osteopenia, osteoporosis**	Adequate diet and vitamin D supplementation	Monitoring of serum vitamin D and bone indices every year. Bone mineral density every year if in treatment; every 3–5 y if normal scans are detected.
